# Characterizing Polarizers with Direct Electrical Readouts

**DOI:** 10.3390/nano16050301

**Published:** 2026-02-27

**Authors:** Longbo Jiao, Lili Xie, Qiuyi Long, Xinchen Li, Yizhi Wu, Weijia Shao, Qingjia Zhou

**Affiliations:** 1 University Engineering Research Center of Advanced Functional Materials and Intelligent Sensing, School of Physical Science and Technology, Guangxi Normal University, Guilin 541004, China; jlb810975@stu.gxnu.edu.cn (L.J.); llxie@stu.gxnu.edu.cn (L.X.); qylong@stu.gxnu.edu.cn (Q.L.); xinchen@stu.gxnu.edu.cn (X.L.); 2 School of Physical Science and Technology, Tiangong University, Tianjin 300387, China; wuyizhi@tiangong.edu.cn

**Keywords:** hot electrons, photoelectric conversion, polarizers, electrical extinction ratio

## Abstract

The polarization of light is recognized as a key physical quantity in describing light-matter interactions. Polarizers are fabricated to selectively respond to light beams with different polarizations. In practice, the operations of a polarization measuring setup require bulky and expensive terminal photodetectors, e.g., a spectrophotometer, to measure the spectral responses associated with different polarizations. To get rid of the unfavorable reliance on conventional photodetectors, polarizers having a Cu-ZnO junction for efficient hot-electron extraction have been designed to give rise to direct electrical readouts. Detailed photoelectric studies reveal that the designed device excites guided-mode resonances with which the device exhibits polarization-dependent energy depositions in absorbable Cu, leading to distinct electrical responses between transverse electric and transverse magnetic polarizations. The electrical extinction ratio increases from 2.7 to 4.4 when the resonance wavelength increases from 767 nm to 869 nm.

## 1. Introduction

Polarization, which describes the oscillation direction of the electric component of a light beam, plays a critical role in determining light-matter interaction. Fundamentally, characterizing the polarization of incident light is a prerequisite for assessing the performance of various photon devices. In practice, polarizers are widely employed to quantitatively evaluate polarization combinations in light beams through selectively responding to electromagnetic waves with different polarization states [1,2,3,4]. In general, optical extinction ratios (ERs) are widely used to describe the polarizer performance. ER is defined as a ratio of transmission or reflection efficiencies upon the polarizers between transverse electric (TE) and transverse magnetic (TM) polarization incidences [5,6]. To enhance ER, metal wire grids that support plasmonic resonances in the case of TM polarizations have been fabricated, exhibiting extremely contrasting spectral responses between TE- and TM-polarized incidences [7,8,9].

In spite of high ERs, polarization measuring systems often require bulky and expensive terminal equipment (e.g., spectrophotometer) to detect different optical powers related to corresponding polarizations, hindering the device-level miniaturizations and integrations [10,11]. In fact, the operations of a variety of optical applications also rely on efficient photodetection for further signal processing and indication. Recently, hot-electron photodetection has been attracting increasing attention due to the unique capabilities of room-temperature operation, ultrafast response time, and tunable working wavelength [12,13,14,15,16]. In order to miniaturize conventional optical sensing systems, metal-semiconductor junctions have been integrated into the plasmonic nanostructured sensors to realize direct electrical readouts by extracting photoexcited electrons due to energy depositions in metals, offering a promising opportunity for optimizing or simplifying other photon systems [17].

Inspired by previously reported hot-electron optical sensors, in this work, a polarizer is designed for electrically characterizing the device performance through efficient hot-electron extraction assisted by a planar Cu-ZnO junction. The designed device has a TiO_2_ grating atop the Cu-ZnO junction to excite the guided-mode resonance (GMR) at an identical wavelength for both TM and TE polarizations. Benefiting from the GMR-induced polarization-dependent reflection efficiencies at 820 nm, the designed polarizer exhibits an optical ER of −6.8 dB. When the GMR wavelength increases from 820 nm to 869 nm, the performance of the polarizer is improved with an optical ER of −23.2 dB. Based on a probability-based photoelectric model, detailed electrical studies were conducted to demonstrate the correlations between the optical absorptions in Cu and responsivities, which are closely related to incident polarizations. The proposed hot-electron polarizers are expected to open the pathway for highly integrated polarization measurement.

## 2. Results and Discussion

**Figure 1:**
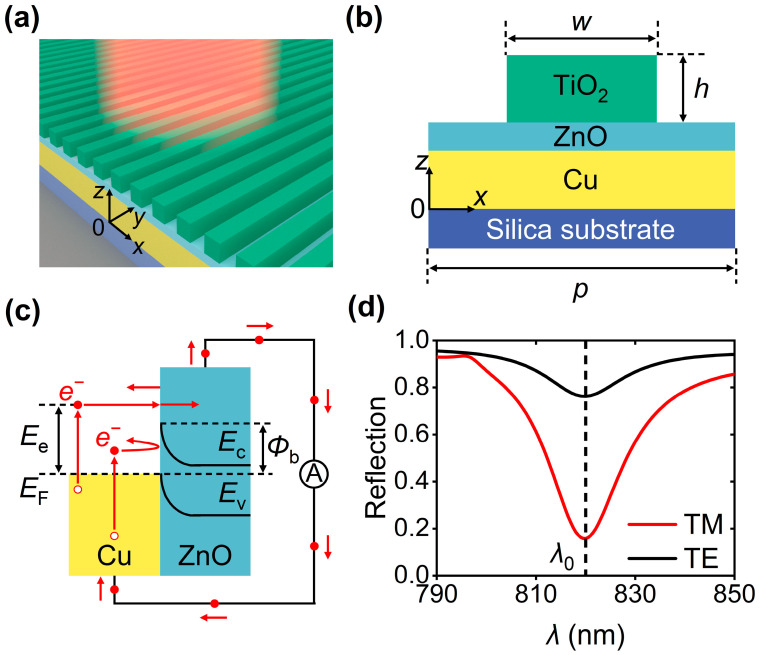
(**a**) Three-dimensional and (**b**) side-view schematics of the designed polarizer. (**c**) Energy band diagram of the Cu-ZnO junction. *E*_c_ and *E*_v_ are the conduction and valence bands of ZnO, respectively. (**d**) Reflection spectra of the designed device under normal illuminations with TM and TE polarizations when *w* = 510 nm and *p* = 788 nm.

As schematically shown in Figure 1a, the designed polarizer is composed of a ZnO layer sandwiched between a TiO_2_ grating and an optically thick Cu layer with a thickness of 200 nm. The whole device is mounted on a silica substrate. Figure 1b shows that the TiO_2_ grating consists of periodically arranged TiO_2_ stripes whose height and width are denoted by *h* and *w*, respectively. The period of the TiO_2_ grating is represented by *p*. In our studies, *w* and *p* are variable, and *h* is fixed to 660 nm. The operations of the designed polarizers rely on efficient photoelectric conversion governed by the hot-electron dynamics in the Cu-ZnO junction, as shown in Figure 1c. When the designed device is illuminated by a normal incidence, the energy depositions in Cu lead to electronic transitions from occupied levels below the Fermi level (*E*_F_) to higher unoccupied levels, generating hot electrons whose energy exceeding *E*_F_ is denoted by *E*_e_. The energy distributions of generated hot electrons significantly deviate from the Fermi-Dirac energy distribution. Therefore, these non-equilibrium hot electrons would undergo ultrafast thermalization in hundreds of femtoseconds. Meanwhile, hot electrons diffuse toward the Cu-ZnO interface with a barrier height (*Φ*_b_) of 0.45 eV [18]. Upon arriving at the Cu-ZnO interface, the below-barrier (*E*_e_ < *Φ*_b_) ones would be blocked by the Schottky barrier; however, the above-barrier (*E*_e_ > *Φ*_b_) ones have a chance to be injected into the ZnO layer, contributing to a steady-state photocurrent. To evaluate the photoelectric responses of the device, we solved Maxwell’s equations to obtain spectral responses in a commercial software (COMSOL Multiphysics 6.3) based on a finite element algorithm. Firstly, we built a geometric model of the unit cell of the designed device with specific structural parameters in the graphic user interface of the software. Secondly, material dispersion data of ZnO, Cu, TiO_2_, and SiO_2_ were put into the software to predict the optical responses precisely. At last, suitable boundary conditions, such as a perfectly matching layer and periodic boundary conditions, were applied to the geometric model to efficiently assess the optical responses, including reflection and absorption spectra of the device. We elaborately adjusted structural parameters (i.e., *w* = 510 nm and *p* = 788 nm) to realize suppressed reflection efficiencies at an identical resonance wavelength (*λ*_0_ = 820 nm) regardless of incident polarizations, as shown in Figure 1d. The reflection efficiency is 0.16 in the case of TM incidence, and the reflection efficiency is up to 0.76 when the incident light is TE-polarized.

**Figure 2:**
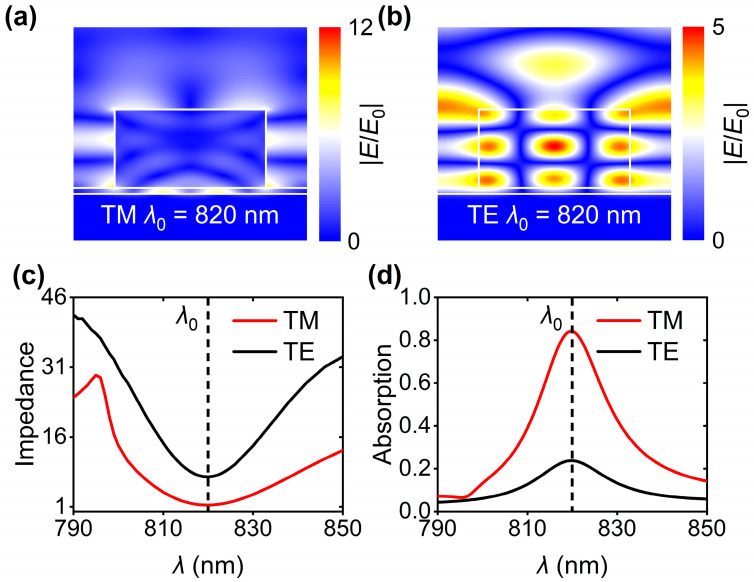
Spatial distributions of the normalized electric fields (|*E*/*E*_0_|) at 820 nm under (**a**) TM- and (**b**) TE-polarized incidences. (**c**) Impedance and (**d**) absorption spectra in the case of TM and TE polarizations. *λ*_0_ is the resonance wavelength. All the simulation results in this figure were obtained when *w* = 510 nm and *p* = 788 nm.

To reveal the underlying physics behind the observed anti-reflection phenomena at 820 nm, the spatial distributions of electric fields (|*E*|) under TM- and TE-polarized illuminations have been plotted, as shown in Figure 2a and Figure 2b, respectively. The electric fields have been normalized by the electric fields (|*E*_0_|) of incident light. It is found that suppressed reflections can be ascribed to the local field enhancement (i.e., |*E*/*E*_0_| > 1). Furthermore, the electric profiles indicate the excitations of GMRs. To gain more insights into the GMR excitations, the wavelength-dependent impedances have been calculated through the method of *S*-parameter, as shown in Figure 2c [19]. Obviously, there are two dips at 820 nm, corresponding to the two reflection dips displayed in Figure 1d. Note that the impedance at 820 nm is close to air impedance (i.e., 1) when the incident light is TM-polarized, leading to distinctly low reflection efficiency. However, TE-polarized incidence produces relatively high device impedance (~7.44) at 820 nm, resulting in a shallow reflection dip with reflectance of 0.76. Due to negligible device transmission, the contrast in reflection efficiencies at 820 nm would give rise to polarization-dependent absorptions in the Cu layer, as shown in Figure 2d. The absorption efficiency reaches 0.84 (0.24) in the case of TM (TE) polarization, probably leading to polarization-related electrical readouts.

**Figure 3:**
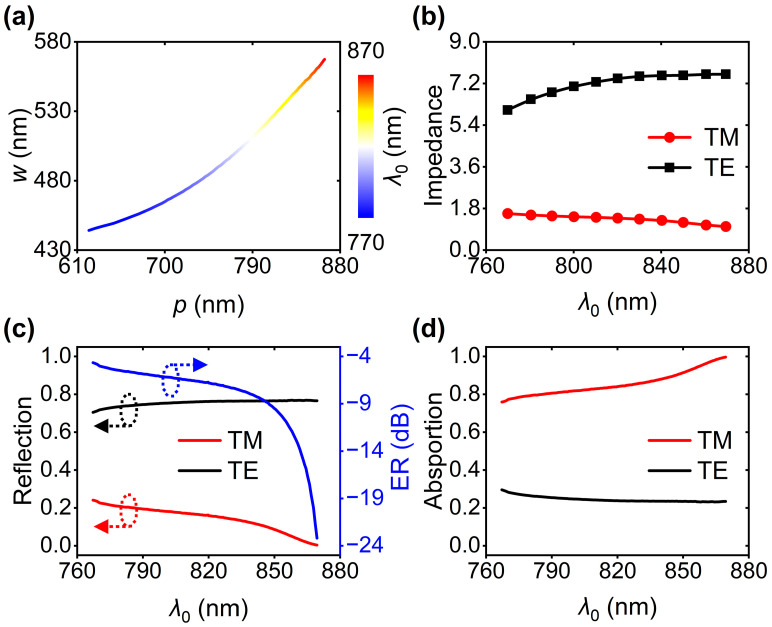
(**a**) Serial combinations of *p* and *w* for exciting GMRs at identical *λ*_0_ in the cases of TM and TE polarizations. *λ*_0_ ranges from 767 nm to 869 nm. The *λ*_0_-dependenet (**b**) impedance, (**c**) reflection and ER, and (**d**) absorption in the Cu layer under TM- and TE-polarized illuminations.

Further, we realized tailored GMRs for both polarizations by exploring serial parameter combinations of *p* and *w*. The colored curve in Figure 3a presents a certain corresponding relationship between variable structural parameters (*p* and *w*) and tunable *λ*_0_ ranging from 767 nm to 869 nm. The dependences of device impedance associated with TM and TE polarizations on *λ*_0_ are also studied, as plotted in Figure 3b. We find that the TM polarization-related impedances are significantly less than those induced by TE-polarized light, leading to distinct reflection efficiencies between TM and TE polarizations, as shown in Figure 3c. In addition, we used a quantity of optical extinction ratio (ER) to describe polarization-dependent spectral responses. ER = −10 × lg(*R*_TE_/*R*_TM_), in which *R*_TM_ and *R*_TE_ are the reflection efficiencies in the cases of TM and TE polarizations, respectively. Optical calculations show that optical ER is −6.8 dB at 820 nm. Furthermore, ER decreases from −4.7 dB to −23.2 dB when *λ*_0_ increases from 767 nm to 869 nm. Provided the polarization-dependent reflections, the absorption efficiencies in the Cu layer are also relevant to the incident polarizations, as shown in Figure 3d. Obviously, compared to the TE-polarized light, TM polarization is favorable for depositing energies in Cu.

**Figure 4:**
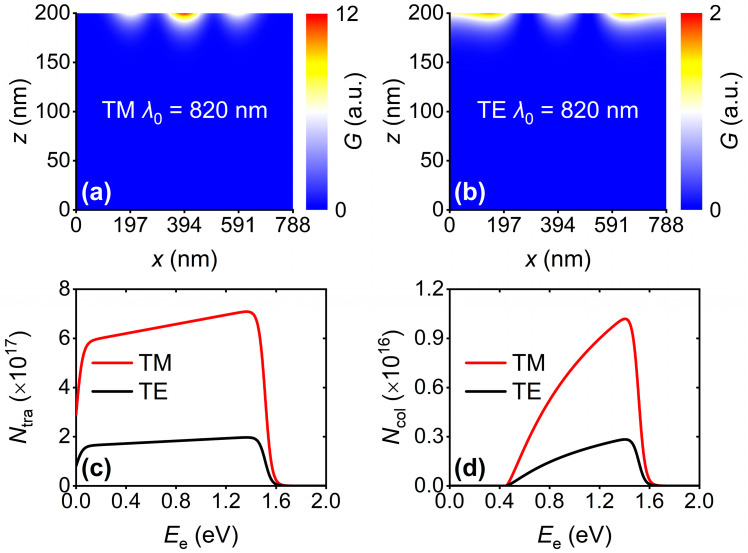
The profiles of the hot-electron generation rates under (**a**) TM- and (**b**) TE-polarized incidences at 820 nm. (**c**) The *E*_e_-dependent flux (*N*_tra_) of hot electrons reaching the Cu-ZnO interface and (**d**) the flux (*N*_col_) of hot electrons that surmount the Cu-ZnO Schottky barrier.

After optical studies, quantitative studies on hot-electron dynamics at 820 nm for different incident polarizations were employed. Firstly, the hot-electron generation rates (*G*) in the cases of TM and TE polarizations were calculated, as depicted in Figure 4a and Figure 4b, respectively. *G* can be expressed as

(1) G=12ωIm(ε)E(x,z)2/hν

where ω is the angular frequency of incident light, Im(*ε*) is the imaginary part of Cu permittivity, and *hν* is the photon energy. It is found that different polarizations give rise to distinct *G* profiles; whereas, almost all hot electrons are generated close to the Cu-ZnO interface, relieving the hot-electron losses in the following transport process. Then, assuming an isotropic hot-electron angular distribution, the *E*_e_-dependent flux (*N*_tra_) of hot electrons arriving at the Cu-ZnO interface can be calculated by

(2) $$N_{\mathrm{tra}}(E_{e})=\int G(x,z)\times D(E_{e})\times P_{\mathrm{tra}}(z,E_{e})dxdz$$

where *D*(*E*_e_) and *P*_tra_ are the hot-electron energy distribution and transport probability, respectively. *D*(*E*_e_) can be evaluated by:

(3) $$D(E_{e})=\frac{\rho (E_{f}-h\nu )f(E_{f}-h\nu )\rho (E_{f})[1-f(E_{f})]}{\int \rho (E_{f}-h\nu )f(E_{f}-h\nu )\rho (E_{f})[1-f(E_{f})]dE_{e}}$$

where *E*_f_ = *E*_e_ + *E*_F_, *ρ*(*E*_f_) is the density of electronic states, and *f*(*E*_f_) is the Fermi-Dirac distribution function. *P*_tra_ can be calculated as

(4) $$P_{\mathrm{tra}}(x,z)=\frac{1}{2}\int_{0}^{\frac{\pi }{2}}\sin\theta \exp(-\frac{d}{\lambda e|\cos\theta |})d\theta$$

where *d* is the distance from the generation position of hot electrons to the Cu-ZnO interface, *θ* is the hot-electron moving angle, and *λ*_e_ is the electronic mean free path in Cu [20]. As shown in Figure 4c, it is revealed that the TM polarization-related *N*_tra_ is prominently larger than that induced by TE-polarized illumination. It is because, compared to TE polarization, TM-polarized incident light induces more energy depositions in the Cu layer. At last, we obtain the flux (*N*_col_) of hot electrons that can contribute to the photocurrent by using the hot-electron injection probability occurring at the Cu-ZnO interface. *N*_col_ is dependent on *E*_e_ and can be written as

(5) $$N_{\mathrm{col}}(E_{e})=\int G(x,z)\times D(E_{e})\times P_{\mathrm{tra}}(z,E_{e})\times P_{\mathrm{inj}}(E_{e})dxdz$$

In addition, *P*_inj_ can be expressed as

(6) $$P_{\mathrm{inj}}=\int_{0}^{k_{\mathrm{ZnO}}}\frac{4\sqrt{\left({k_{\mathrm{Cu}}}^{2}-{k_{y}}^{2})({k_{\mathrm{ZnO}}}^{2}-{k_{y}}^{2}\right)}}{\sqrt{\left({k_{\mathrm{Cu}}}^{2}-{k_{y}}^{2}\right)}+\sqrt{\left({k_{\mathrm{ZnO}}}^{2}-{k_{y}}^{2}\right)}}\frac{k_{y}dk_{y}}{2k_{\mathrm{Cu}}\sqrt{\left({k_{\mathrm{Cu}}}^{2}-{k_{y}}^{2}\right)}}$$

where *k*_ZnO_ and *k*_Cu_ are the hot-electron momenta in ZnO and Cu [21,22], respectively. As shown in Figure 4d, *N*_col_ = 0 when *E*_e_ < 0.45 eV for both polarizations. When *E*_e_ is larger than barrier height, *N*_col_ is in direct proportion to *E*_e_. But *N*_col_ rapidly decreases to 0 when *E*_e_ > ~1.51 eV. It is because the energies of almost hot electrons are less than the photon energy (1.51 eV).

**Figure 5:**
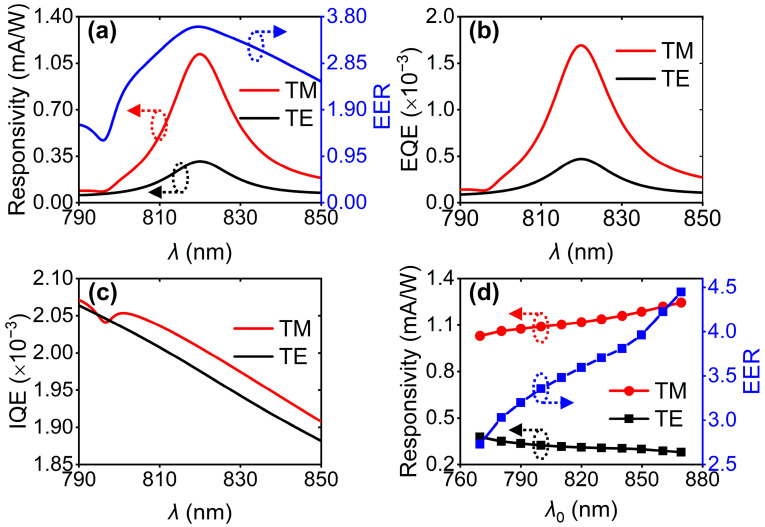
(**a**) Responsivity and EER as a function of wavelength. Calculated wavelength-dependent (**b**) EQE and (**c**) IQE. (**d**) Responsivity and EER spectra relevant resonance wavelength ranged from 767 nm to 869 nm. Both TM and TE polarizations are considered for the above calculations.

Based on the above-mentioned hot-electron dynamic analysis, we can obtain the responsivity spectra by integrating *N*_col_ over an *E*_e_ scope ranging from 0 eV to 2.0 eV. As shown in Figure 5a, one can see that the device responsivity at 820 nm in the case of TM-polarized incidence is up to 1.61 mA/W; however, the responsivity is only 0.45 mA/W when the designed polarizer is under TE-polarized illumination with a wavelength of 820 nm. The distinct electrical responses under different polarizations can be used to electrically characterize the performance of the designed polarizer. Furthermore, we defined a quantity of electrical extinction ratio (EER) that is expressed as a ratio of responsivity between TM and TE polarizations [23], as shown by the blue line shown in Figure 5a. As expected, EER has a peak value of 3.6 at 820 nm. To further analyze the polarization-dependent responsivity spectra, the dependence of the external quantum efficiency (EQE) on the wavelength has been studied, as shown in Figure 5b. Obviously, both EQE spectra under two polarizations have a peak at 820 nm, and the TM-related EQE is larger than that induced by TE-polarized illumination. It is known that EQE convolutes the effects of energy absorption with the subsequent electronic processes that occur within the Cu-ZnO junction; therefore, the internal quantum efficiency (IQE) spectra corresponding to TM and TE polarizations are plotted, as depicted in Figure 5c. It is revealed that both IQEs generally decrease with wavelength due to the reduction in the proportion of above-barrier hot electrons with the increase in wavelength. Finally, we focused on the electrical responses and EER at *λ*_0_, as shown in Figure 5d. Basically, the responsivity in the case of TM (TE) polarization increases (decreases) when *λ*_0_ increases from 767 nm to 869 nm, resulting in a growth of EER from 2.7 to 4.4. It is because the TM (TE) polarization-induced absorption in Cu increases (decreases) with the increase in *λ*_0_, as shown in Figure 3d.

## 3. Conclusions

In summary, we present a design of polarizers whose performances can be assessed by direct electrical readouts, rather than through optical responses. The designed device is composed of a planar Cu-ZnO junction capped with a TiO_2_ grating. Optical studies reveal the polarizer supports GMR excitations at identical resonance wavelengths but with distinct reflection efficiencies related to incident polarizations, which can be harnessed to describe polarization combinations in incident light. Further optical calculations show tunable optical ER from −4.7 dB to −23.2 dB when GMR wavelengths increase from 767 nm to 869 nm. In terms of EER for assessing the designed polarizer, EER varies in the range from 2.5 to 4.5 for the considered range of resonance wavelength.

## Data Availability

The raw data supporting the conclusions of this article will be made available by the authors on request.
